# Depiction rate of feeding arteries of renal cell carcinoma on four-dimensional computed tomography angiography

**DOI:** 10.1007/s11604-024-01538-y

**Published:** 2024-02-23

**Authors:** Kazuaki Munetomo, Yusuke Matsui, Koji Tomita, Mayu Uka, Noriyuki Umakoshi, Takahiro Kawabata, Yusuke Morimitsu, Toshihiro Iguchi, Takao Hiraki

**Affiliations:** 1 Department of Radiology, Graduate School of Medicine, Dentistry and Pharmaceutical Sciences, Okayama University, 2-5-1 Shikata-Cho, Kita-Ku, Okayama, 700-8558 Japan; 2 Department of Radiology, Okayama University Hospital, Okayama, Japan; 3 Department of Radiology, Faculty of Medicine, Dentistry and Pharmaceutical Sciences, Okayama University, Okayama, Japan; 4 Department of Radiological Technology, Okayama University Hospital, Okayama, Japan; 5 Department of Radiological Technology, Faculty of Health Sciences, Okayama University, Okayama, Japan

**Keywords:** Four-dimensional computed tomography, Computed tomography angiography, Kidney, Feeding artery, Transcatheter arterial embolization

## Abstract

**Purpose:**

To retrospectively evaluate the depiction rate of feeding arteries in biopsy-proven clear cell renal cell carcinoma (CCRCC) on four-dimensional computed tomography angiography (4D-CTA) images.

**Materials and methods:**

This study included 22 patients with 22 CCRCC and 30 feeding arteries treated with transcatheter renal artery embolization. The depiction rate of the feeding arteries on preprocedural 4D-CTA was evaluated. Images were acquired by 320-row multi-detector computed tomography (CT) 15‒36 s after starting to inject a contrast agent (600 mg/kg iodine) intravenously into patients at 2.1 s intervals (11 phases). Two board-certified radiologists retrospectively assessed the feeder depiction rate in all 11 phases with reference to the procedural images as the gold standard. Discrepancies were resolved by consultation with a third radiologist.

**Results:**

Among the feeders, 11 (36.7%) were segmental or lobar, and 19 (63.3%) were interlobar or arcuate arteries. The feeder depiction rate was the highest (25 [83.3%] of 30) in the 5th phase (delay, 23.4 s) where the gap in contrast enhancement between the renal artery and cortex was the largest. This was followed by the 6th (23 [76.7%] of 30), 4th (22 [73.3%] of 30]), and 7th (21 [70.0%] of 30) phases. The overall rate of depicting feeding arteries in the 11 phases of 4D-CTA was 28 (93.3%) of 30.

**Conclusions:**

The depiction rate of CCRCC feeding arteries including lobar or smaller artery branches by 4D-CTA was favorable. The feeding arteries were optimally visualized during the phase with the largest contrast gap between the renal artery and cortex.

## Introduction

Transcatheter renal artery embolization (TRAE) is widely used to treat renal tumors [1–3]. In recent years, this procedure has been performed before percutaneous cryoablation or radiofrequency ablation for renal cancers with the aim of tumor marking, enlarging the ablative area, and decreasing procedure-related bleeding [4–6].

Preprocedural imaging is crucial for procedural planning and successful embolization of renal tumors [3]. Identifying tumor-feeding arteries before transcatheter embolization may reduce the amount of contrast medium and the radiation dose required during the procedure [7, 8]. However, it is not always easy to identify feeding arteries before selective TRAE procedures using conventional computed tomography angiography (CTA). Because the renal cortex and veins are considerably enhanced within the early phase after contrast administration, the optimal time window for clearly visualizing the renal artery is generally short [9]. Furthermore, the optimal scan timing for detecting feeding arteries might differ among patients due to variations in the cardiovascular dynamics [10].

Area-detector computed tomography (CT) has been put into practical use, enabling a large-volume scan covering an entire organ with a single rotation [11]. A 320-row detector CT enables continuous imaging of a 16-cm wide area during contrast administration and can provide time-resolved (four-dimensional [4D]) CTA images covering the whole kidney. There have been reports on the usefulness of 4D-CTA in assessing vasculature in neurovascular disorders [11–13]. While studies on this technique in an abdominal setting have been limited, we postulated that 4D-CTA might be advantageous in the detailed evaluation of renal vasculature, making it possible to select the images of an optimal time phase for visualizing tumor-feeding arteries from multiphasic image data with high temporal resolution.

The purpose of this study was to retrospectively evaluate the depiction rate of feeding arteries in clear cell renal cell carcinoma (CCRCC) using preprocedural 4D-CTA and to determine the optimal timing for visualizing feeding arteries.

## Materials and methods

This study was retrospectively conducted at a single center with the approval of the institutional review board (approval number: KEN2201-031). Written informed consent was waived, and opt-out consent was obtained for retrospective analysis of innominate patient data.

### Study population

**Fig. 1:**
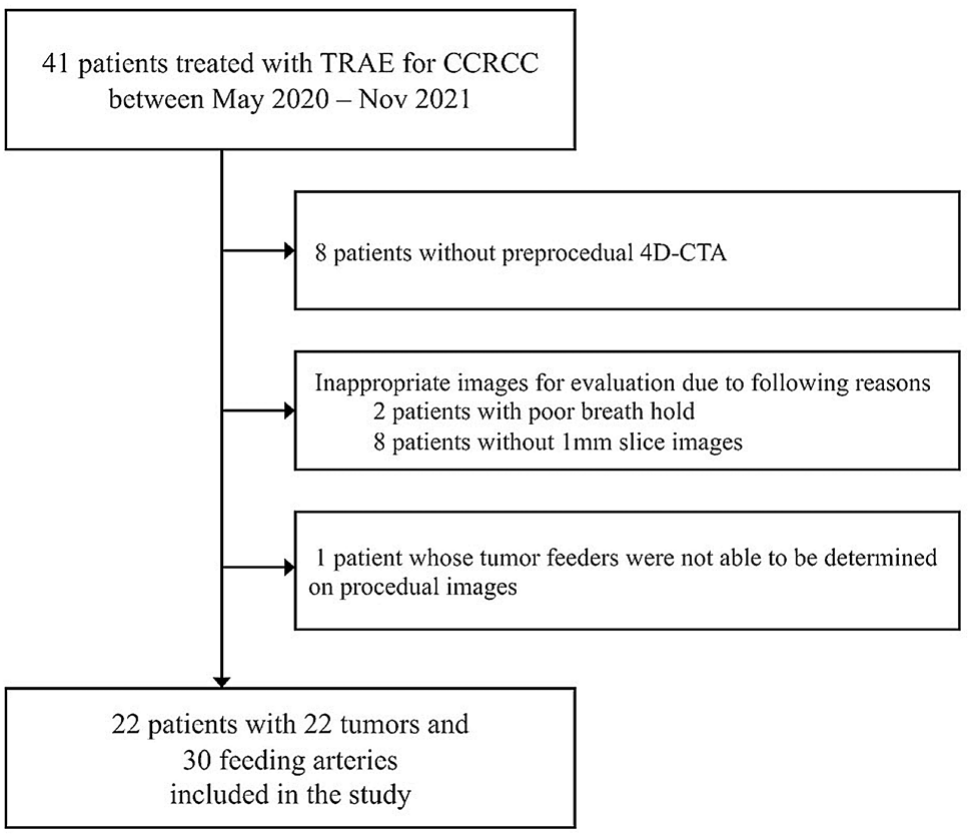
Patient flow chart. *TRAE* transcatheter renal artery embolization; *CCRCC* clear cell renal cell carcinoma, *4D-CTA* four-dimensional computed tomography angiography

This study included patients who underwent TRAE for biopsy-proven CCRCC between May 2020 and November 2021. All TRAE procedures were performed before cryoablation of CCRCC to enhance safety and treatment efficacy [4–6]. Renal 4D-CTA was performed within 4 months before TRAE for procedure planning. Patients who met at least one of the following criteria were excluded: (i) 4D-CTA images did not follow the standard institutional protocol described below; (ii) 4D-CTA images were not evaluable due to poor breath-hold or absence of 1-mm slice images of all phases; and (iii) tumor feeders could not be determined on procedural images. Figure 1 shows the flowchart of patient selection. A total of 41 patients underwent TRAE for biopsy-proven CCRCC. Of these, 19 were excluded due to missing preprocedural 4D-CTA data (n = 8), images unsuitable for evaluation due to poor breath holding (n = 2), missing 1 mm slice data of all phases (n = 8), or inability to identify tumor feeders on procedural images (n = 1). Thus, we analyzed data from 22 patients (male, n = 16; female, n = 6; mean age ± standard deviation [SD], 67.2 ± 9.5 y) with 22 tumors (mean diameter ± SD, 20.0 ± 7.1 mm). Fourteen and eight tumors were respectively nourished by single and dual feeders. Therefore, we analyzed 30 feeding arteries. Table 1 summarizes the characteristics of the patients and tumors.

**Table 1:** Characteristics of 22 patients and 22 tumors

Variable		Value
Patient		
Age (years)	Mean ± SD	67.2 ± 9.5
Sex	Man/woman	16/6
CT scan position	Supine/prone	6/16
Single kidney	Yes/no	6/16
eGFR (mL/min/1.73m^2^)	Mean ± SD	62.8 ± 16.5
BMI	Mean ± SD	24.2 ± 2.6
Tumor		
Size (mm)	Mean ± SD	20.0 ± 7.1
Laterality	Left/Right	12/10
Position	Exophytic/Parenchymal	15/7
Number of feeding artery	Single/Dual	14/8

### Renal 4D-CTA

Renal 4D-CTA images were acquired using an area-detector CT with a 0.5-mm × 320-row detector (Aquilion ONE; Canon Medical Systems, Otawara, Japan). Patients were placed in a prone or supine position with the upper extremities elevated over the head. The prone position was applied when necessary to plan the cryoprobes’ trajectory [14]. All patients were injected with 600 mg/kg of nonionic iodine contrast medium at a maximum injection rate of 5 mL/s over 22 s through a 20-gauge angiocatheter in an upper extremity vein. A total of 11 intermittent CT series was obtained using a 160 mm volume scan without moving the table and gantry every 2.1 s from 15.0 s to 36.0 s after the start of contrast injection while the patient was in expiratory breath-hold. The rotation time was set at 0.275 s. The tube voltage was 100 kV, and an automatic exposure control (AEC) was used with its standard deviation (SD) set at 35. Subsequently, conventional renal parenchymal phase (delay time: 63 s) and excretory phase (delay time: 4 min) images were acquired using a helical scan. The CT images were reconstructed using model-based iterative reconstruction algorithms (FIRST Body Sharp Mild; Canon Medical Systems). Multiplanar reconstruction (MPR) images were prepared for analysis, including 1-mm axial, sagittal, and coronal slices. In addition, maximum intensity projection (MIP) images were reconstructed. The volume CT dose index (CTDIvol) and dose-length product (DLP) for 4D-CTA were recorded.

### Transcatheter renal artery embolization

TRAE was performed in an angiography suite equipped with an angio-CT system consisting of a CT scanner (Aquilion ONE or Aquilion CX; Canon Medical Systems) and an angiographic C-arm (Alphenix or Infinix Celeve-I; Canon Medical Systems) by board-certified interventional radiologists or radiology trainees under the supervision of board-certified interventional radiologists. The renal artery was selected using a 4-Fr catheter via the transfemoral approach, and transcatheter renal CTA and digital subtraction angiography (DSA) was performed from the main trunk of the renal artery. We applied DSA primarily to seek tumor staining and involved vessels, and transcatheter CTA images were also scrutinized when a feeding artery was obscure on DSA images. A microcatheter was then advanced into the feeding arteries. After confirming tumor staining by selective angiography, ethiodized oil was injected for embolization. In addition, ethanol, gelatin sponges, and coils were used as necessary at the discretion of operators. The procedure was completed after DSA confirmed the absence of tumor stain. Ethiodized oil deposition throughout the tumor was confirmed using unenhanced CT images in all patients. The safety margin of the embolized area was not required because TRAE was an adjunct to cryoablation, primarily for tumor marking, and was not intended for tumor eradication.

### Image analysis

Time-density curves (TDCs) were created by drawing 2-mm circular regions of interest on the distal portion of the renal artery trunk and the renal cortex in the same slice of 1-mm axial 4D-CTA images. Thereafter, differences in CT values between the renal artery and cortex in each phase were calculated and plotted. We defined a feeding artery as the renal artery branch where the microcatheter was placed and embolic material was injected during the TRAE procedure. The feeding arteries were classified into two groups according to their location as follows: (i) those surrounded by renal sinus fat (segmental or lobar arteries), and (ii) those in the renal parenchyma (interlobar or arcuate arteries) [1]. Two board-certified radiologists with 16 and 12 years of experience, respectively, independently evaluated the 11 phases of 4D-CTA images in all patients and determined whether feeding arteries were identified in each phase, with reference to procedural DSA and CTA as the gold standard. Axial sections of 1 mm slices were primarily assessed and MPR and MIP images were assessed as required. Discrepancies were resolved by a third radiologist with 17 years of experience.

### Statistical analysis

Continuous and categorical variables related to the patients, tumors, and feeding arteries were summarized as means ± SDs and raw numbers with percentages (n%), respectively. CTDIvol (mGy) and DLP (mGy.cm) of 4D-CTA were also summarized as means ± SDs. The depiction rate of the feeding arteries was calculated as the proportion (%) that were visible in each phase among the total number of feeding arteries. We then calculated overall depiction rates as the number of feeding arteries found in any of the 11 phases of 4D-CTA as the numerator and the total number of feeding arteries as the denominator. Data were analyzed using Excel for Mac v.16.67 (Microsoft Corp., Redwood, WA, USA).

## Results

**Fig. 2:**
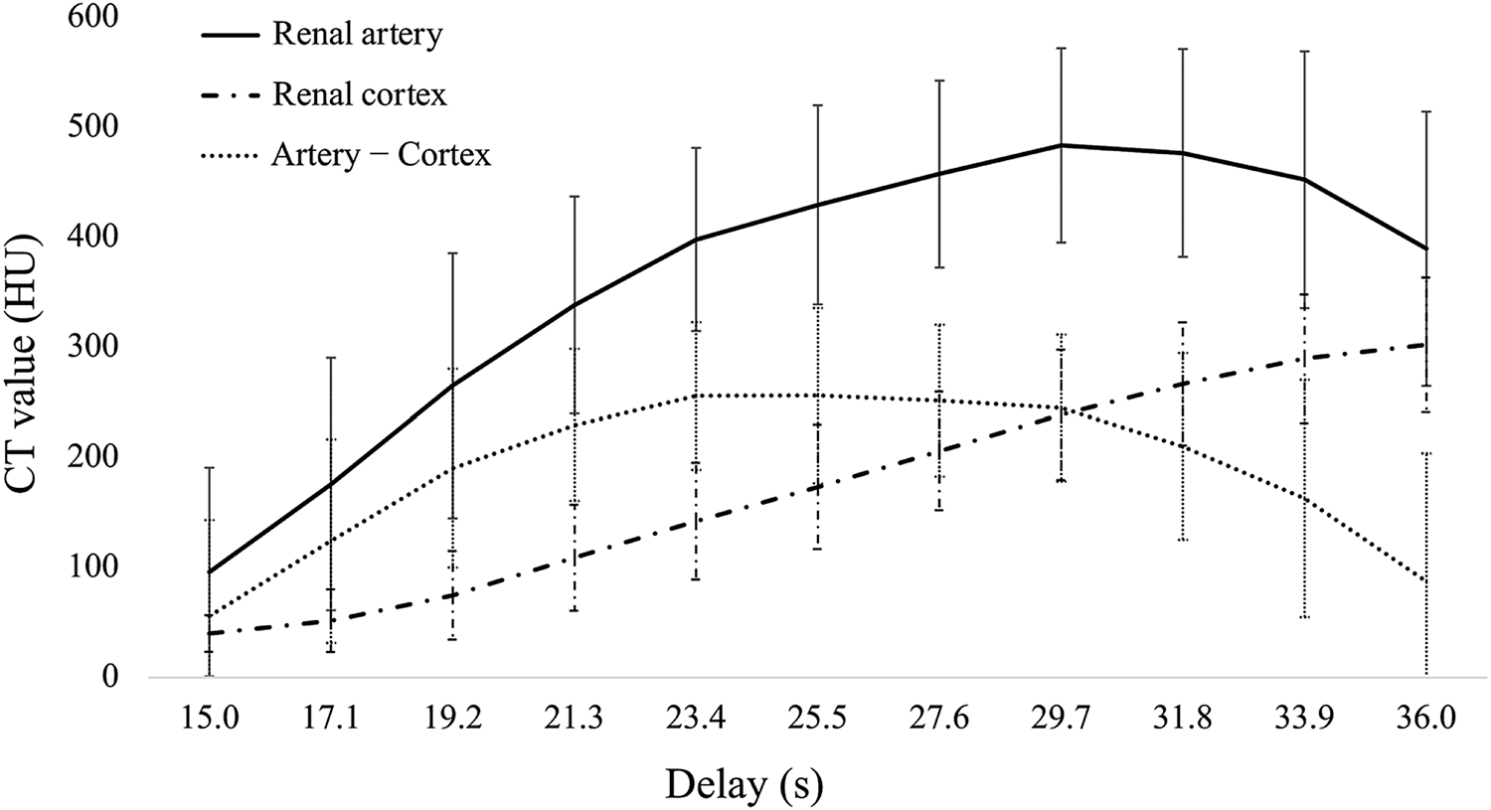
Time density curves of renal artery and cortex. CT values were measured on 1-mm axial images by placing 2-mm circular regions of interest on the distal portion of the renal artery trunk and cortex in the same slice to generate time density curves. *CT* computed tomography, *HU* Hounsfield units

The TDCs of the renal artery and cortex in the 4D-CTA images are shown in Fig. 2. The mean CT value of the renal artery peaked (483.7 ± 88.4 Hounsfield units [HU]) at a delay time of 29.7 s (8th phase). The mean CT value of the renal cortex continuously increased between delay times of 15.0 s and 36.0 s. The difference in the CT values between the renal artery and cortex (artery − cortex) was largest when the delay was 23.4 s (5th phase). The CTDIvol and DLP for 4D-CTA were 32.9 ± 3.2 mGy and 526.7 ± 50.9 mGy.cm, respectively.

**Fig. 3:**
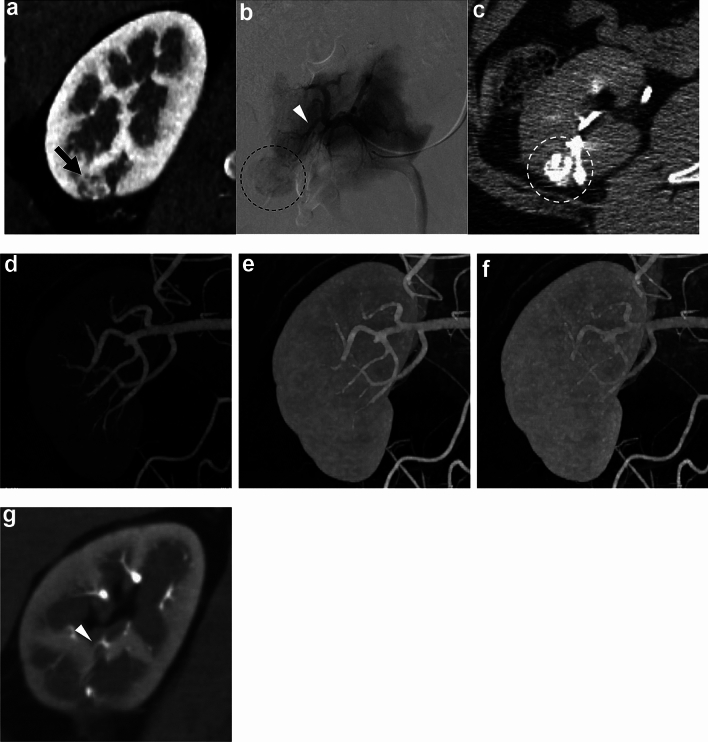
Preprocedural renal 4D-CTA and procedural DSA images from a patient with CCRCC with a single feeding artery. **a** Preprocedural coronal CT image shows CCRCC (14 mm) in the right kidney (black arrow). **b** Digital subtraction arteriography via microcatheter in segmental artery shows feeding artery (white arrowhead) and tumor stain (black dotted circle). The microcatheter was advanced to the position indicated by white arrowhead to embolize the feeding artery. **c** Axial CT image immediately after embolization shows that ethiodized oil is deposited throughout the tumor (white dotted circle). **d**‒**f** Coronal maximum intensity projection images of the 4th (**d**), 10th (**e**), and 11th (**f**) phases (delays 21.3, 33.9, and 36.0 s, respectively) of 4D-CTA show various degrees of renal artery branches. The 4th phase corresponds to that where the difference in contrast enhancement is the largest between renal artery and cortex in this patient, whereas renal artery enhancement is maximal in the 10th phase. Peripheral branches are most obvious in the 4th phase. **g** Coronal image of the 4th phase shows the corresponding feeding artery in renal parenchyma (white arrowhead). *4D-CTA* four-dimensional computed tomography angiography, *CCRCC* clear cell renal cell carcinoma, *DSA* digital subtraction arteriography

Among 30 feeding arteries, 11 (36.7%) were segmental or lobar, and 19 (63.3%) were interlobar or arcuate arteries. We injected ethiodized oil from the segmental arteries without the need for deeper catheterization in two patients because their tumors (33 or 28 mm) were fed by multiple tiny arterial branches from the segmental artery. The mean sizes of tumors with segmental or lobar and interlobar or arcuate feeding arteries were 23.3 ± 8.0 and 18.6 ± 6.6 mm, respectively. Table 2 shows the image analysis findings of feeding arteries in each phase. The depiction rate varied by phase and was the highest (25 [83.3%] of 30) in the 5th phase where the gap in contrast enhancement between the renal artery and cortex in the TDC was the largest (Fig. 2). This was followed by the 6th, 4th, and 7th phases with rates of 23 (76.7%), 22 (73.3%), and 21 (70.0%) of 30, respectively. Four of the five feeding arteries that could not be identified in the 5th phase were interlobar or arcuate. Three of the five were evident in other phases, whereas the other two were not found in any phases. Thus, the overall depiction rate of feeding arteries in 4D-CTA was 28 (93.3%) of 30. The two arteries that were not found in any phases fed the smallest tumor (9 mm). Figure 3 shows preprocedural renal 4D-CTA and procedural DSA images from a patient.

**Table 2:** Depiction of 30 feeding arteries of 22 CCRCC in each phase of 4D-CTA

Patient No.	Feeder No.	Phase No. [Delay (s)]											
		1^st^ [15.0]	2^nd^ [17.1]	3^rd^ [19.2]	4^th^ [21.3]	5^th^ [23.4]	6^th^ [25.5]	7^th^ [27.6]	8^th^ [29.7]	9^th^ [31.8]	10^th^ [33.9]	11^th^ [36.0]	All phases
1	1	–	–	–	–	D	D	D	D	D	D	D	D
2	1	–	–	D	D	D	–	–	–	–	–	–	D
3	1	–	–	–	–	D	D	D	D	D	D	D	D
4	1	–	–	D	D	D	D	D	–	–	–	–	D
5	1	–	D	D	D	D	D	D	D	D	D	–	D
6	1	–	–	–	D	D	D	D	D	D	D	D	D
	2	–	–	–	–	D	–	–	–	–	–	–	D
7	1	–	–	–	D	D	D	–	–	–	–	–	D
8	1	-	D	D	D	D	-	–	–	–	–	–	D
9	1	–	–	–	–	D	D	–	–	–	–	–	D
10	1	–	–	–	–	–	–	–	–	–	–	–	–
	2	–	–	–	–	–	–	–	–	–	–	–	–
11	1	–	–	D	D	D	D	D	D	–	–	–	D
	2	–	–	–	–	–	D	D	–	–	–	–	D
12	1	–	–	–	D	D	D	D	D	D	D	D	D
	2	–	–	–	D	D	D	D	D	D	D	D	D
13	1	–	–	D	D	D	D	D	D	D	–	–	D
14	1	–	–	–	D	D	D	D	D	D	D	D	D
15	1	D	D	D	D	D	D	D	D	D	–	–	D
	2	–	D	–	D	–	–	–	D	D	–	–	D
16	1	–	–	–	D	D	D	D	D	–	–	–	D
17	1	–	–	–	D	D	D	D	D	–	–	–	D
18	1	–	–	D	D	D	D	D	D	D	D	D	D
	2	–	–	D	D	D	D	D	D	D	D	–	D
19	1	–	–	–	–	D	D	D	–	–	–	–	D
	2	–	–	–	D	–	–	–	–	–	–	–	D
20	1	–	–	–	D	D	D	D	D	D	D	–	D
	2	–	–	–	D	D	D	D	D	D	D	–	D
21	1	D	D	D	D	D	D	D	D	D	–	–	D
22	1	–	–	–	D	D	D	D	D	D	D	D	D
Depicted feeder (n)		2	5	10	22	25	23	21	19	16	12	8	28
Depiction rate (%)		6.7	16.7	33.3	73.3	83.3	76.7	70.0	63.3	53.3	40.0	26.7	93.3

*CCRCC* clear cell renal cell carcinoma, *4D-CTA* four-dimensional computed tomography angiography, *D* depicted, *-* not depicted

## Discussion

The overall depiction rate of feeding arteries of CCRCC on 4D-CTA images was favorable (93.3%). Among 11 phases, the depiction rate was the highest (83.3%) in the 5th phase (delay, 23.4 s). Interlobar or arcuate arteries in the renal parenchyma tended to be less visible than segmental or lobar arteries surrounded by renal sinus fat.

Preprocedural CTA contributes to the identification of target vessels, which enables a reduction in contrast volume and fluoroscopy time during the procedure [7, 8, 15]. Although the advantages of preprocedural CTA have been shown in transcatheter arterial chemoembolization (TACE) for hepatocellular carcinoma (HCC) and prostatic artery embolization [7, 8, 15], CTA before TRAE has not been discussed in detail in the literature. Most studies on renal CTA have evaluated renal artery anatomy before surgery, such as partial nephrectomy or renal transplantation [16–18]. The target vessels evaluated in those studies were the renal artery trunk or segmental arteries. The detection rate of the segmental-level feeding arteries of RCCs in CTA ranged from 84 to 100% [16–18]. However, data about smaller renal artery branches (lobar or more distal-level branches) that require catheterization during selective TRAE procedures are scant. Since the visualization of distal-level feeding arteries should be more challenging because of their small diameter, the overall depiction rate of 93.3%, including those arteries by 4D-CTA, appears promising, suggesting that preprocedural 4D-CTA is useful for planning TRAE.

The 4D-CT images can be obtained using CT systems equipped with multi-row detectors ranging from 128- to 320-rows, which allow continuous or short-interval sequential scanning of a specific region for a period of time [12]. The 4D-CTA method involves a 4D-CT scan while administering contrast media to analyze the detailed vasculature and hemodynamics in various organs and lesions [7, 11–13]. In neurovascular settings, Willems et al. showed that 4D-CTA enables highly accurate identification of the feeding arteries of intracranial arteriovenous malformations [13]. In abdominal settings, Albrecht et al. investigated the impact of 4D-CTA before TACE for HCC [7]. They omitted stepwise extensive DSA during TACE procedures by introducing preprocedural 4D-CTA to detect tumor feeders [7]. This change in the workflow helped 4D-CTA to reduce the volume of intra-arterial contrast media by two-thirds and the radiation dose to operators by half during TACE [7]. The authors noted that 4D-CTA ensures sufficient contrast in the vessels owing to the inclusion of multiple time points, unlike conventional CTA which may miss the optimal arterial contrast timing depending on the examiner and patient [7]. The present study supports this hypothesis: renal 4D-CTA enabled post-processing selection of the optimal phase to visualize the feeding arteries of CCRCC.

Because the transit time of contrast media through the renal circulation is short, the renal vein is enhanced soon after the renal artery is enhanced [19]. Furthermore, during this brief period, the renal cortex and veins are intensely enhanced, obscuring the depiction of the small peripheral renal artery branches [9]. Small renal arterial branches cannot always be visualized in the renal artery phase of conventional dynamic CT [20]. In 4D-CTA with high temporal resolution, feeding arteries were favorably identified in specific phases where the CT values between the renal artery and cortex largely differed. Combining multiple phases further improved the identification, indicating that optimal timing for visualizing feeders varies among patients. Therefore, ensuring the imaging at the optimal time for visualizing feeding arteries is considered difficult by conventional CTA in which the delay time is fixed. Bolus tracking might help to optimize the timing of image acquisition and obtain an adequate renal arterial phase [21]. However, the present findings indicate that this is insufficient to reveal small feeding arteries because phases that are ideal and those with maximum arterial enhancement do not necessarily correspond.

Because our 4D-CTA protocol involved 11 sequential scans, we were concerned about high-radiation doses. Therefore, we configured scan parameters to minimize the radiation dose (tube voltage of 100 kV and AEC for tube current with SD35) while maintaining acceptable image quality, thus resulting in a DLP of 527 mGy.cm for 4D-CTA. The DLP value appears acceptable, compared with diagnostic reference levels of abdominopelvic contrast CT (600–1000 mGy.cm) in several countries [22]. Our results showed that the feeding arteries were most obvious during the 4th‒7th phases (delay, 21.3‒27.6 s). Thus, further dose reduction without impairing the ability to reveal feeding arteries might be achieved by limiting 4D-CT image acquisition to this time range.

In the present study population, cryoablation was planned and prepared based on the findings of 4D-CTA images. Treatment was planned in the same way as that based on conventional contrast-enhanced CT findings without a separate examination that causes extra radiation exposure. Thus, 4D-CTA did not confer any disadvantages in this respect.

This study had several limitations. It was a single-center study with a small number of patients. Renal tumors of histological subtypes other than CCRCC were excluded. The included tumors were relatively small in diameter and had a single or dual feeding artery. The present study retrospectively examined whether feeding arteries embolized during the procedure were visible on preprocedural 4D-CTA images. Therefore, a prospective study is needed to thoroughly evaluate the ability of 4D-CTA to detect feeding arteries. This would enable confirmation of whether or not potential feeders found on preprocedural 4D-CTA are true feeders using selective DSA from the corresponding position during the procedure. Because this study was still in the preliminary phase of assessing preprocedural 4D-CTA, it did not include changes in TRAE procedures such as omitting stepwise DSA. Thus, the contribution of 4D-CTA to a reduction in the contrast amount and radiation exposure during TRAE could not be examined. A comparative study is warranted to prove that preprocedural 4D-CTA contributes to such reductions. The area-detector CT system was not the latest version, so we had no access to the most recent deep-learning image reconstruction algorithms. We therefore applied model-based iterative reconstruction algorithms. Deep-learning reconstruction might improve image quality while minimizing radiation exposure [23].

## Conclusion

Feeding arteries of CCRCC, including lobar or smaller artery branches, were favorably depicted on 4D-CTA images. They were optimally visible during the phase with the largest contrast gap between the renal artery and cortex.
